# The Glutathione S-Transferase P1 341C>T Polymorphism and Cancer Risk: A Meta-Analysis of 28 Case-Control Studies

**DOI:** 10.1371/journal.pone.0056722

**Published:** 2013-02-21

**Authors:** Sheng-xin Huang, Fei-xiang Wu, Min Luo, Liang Ma, Ke-feng Gao, Jian Li, Wen-juan Wu, Shan Huang, Qi Yang, Ke Liu, Yin-nong Zhao, Le-qun Li

**Affiliations:** 1 Department of Hepatobiliary Surgery, Affiliated Tumor Hospital of Guangxi Medical University, Nanning, People's Republic of China; 2 Department of Experiment, Affiliated Tumor Hospital of Guangxi Medical University, Nanning, People's Republic of China; 3 Department of Thoracic Surgery, Affiliated Tumor Hospital of Guangxi Medical University, Nanning, People's Republic of China; 4 Department of Gynecologic Oncology, Affiliated Tumor Hospital of Guangxi Medical University, Nanning, People's Republic of China; Tor Vergata University of Rome, Italy

## Abstract

**Background:**

GSTP1, which is one major group of the glutathione S-transferase family, plays an important role in the metabolism of carcinogens and toxins, reducing damage of DNA as a suppressor of carcinogenesis. The 341C>T polymorphism of the GSTP1 has been implicated in cancer risk through cutting down its metabolic detoxification activities. However, results from previous studies remain conflicting rather than conclusive. To clarify the correlation and provide more statistical evidence for detecting the significance of 341C>T, a meta-analysis was conducted.

**Methodology/Principal Findings:**

The relevant studies were identified through searching of PubMed, Embase, ISI Web of Knowledge and China National Knowledge Infrastructure in August 2012, and selected based on the established inclusion criteria for publications, then a meta-analysis was performed to quantitatively summarize the association of GSTP1 341C>T polymorphism with cancer susceptibility. Stratified analyses were employed to identify the source of heterogeneity. Publication bias was evaluated as well as sensitivity analysis. Based on 28 case-control studies with 13249 cases and 16798 controls, the pooled results indicated that the variant genotypes significantly increased the risk of cancer in homozygote comparison (TT versus CC: *P* = 0.012, OR = 1.40, 95% CI: 1.08–1.81, *P*_het._ = 0.575), and recessive model (TT versus CT/CC: *P* = 0.012, OR = 1.40, 95% CI: 1.08–1.81, *P*_het._ = 0.562). This was confirmed when stratified analyses were conducted according to ethnicity, source of control, matched control, quality score and cancer types. Moreover, significantly increased risk of cancer was also found in lung cancer (heterozygote comparison and dominant model). The stability of these observations was confirmed by a sensitivity analysis. Begger's funnel plot and Egger's test did not reveal any publication bias.

**Conclusions/Significance:**

This meta-analysis suggests that the GSTP1 341C>T polymorphism may contribute to genetic susceptibility to cancer, especially to lung cancer, and in Asian population. Nevertheless, additional well-designed studies focusing on different ethnicity and cancer types are needed to provide a more exact and comprehensive conclusion.

## Introduction

Approximately 12.7 million new cases of cancer, and 7.6 million cancer-related deaths occurred in 2008, which indicated global burden of cancer continues to aggravate [1]. Prevention and diagnosis of cancer have become one of the most important challenges of the world. Potent markers for screening high-risk populations are urgently needed for early detection and preventive actions. The development of cancer is a complicated biological process, which is involved with multi-gene mutations and multiple factors, while the individual hereditary background may be the primarily determined risk of cancer susceptibility. The interaction between genetic susceptibility genes with environmental factors and metabolism dysfunction throughout the body plays an important role in the development of cancer [2]. The glutathione S-transferase family (GSTs), which are composed of four major groups including GSTA (α), GSTM1 (μ), GSTT1 (θ), and GSTP1 (π), are phase II detoxifying enzymes that catalyze a variety of reduced glutathione-dependent reactions with electrophilic substrates [3]. Based on the catalytic activity, GSTs metabolize carcinogens and toxins to reduce damage of DNA as a suppressor of carcinogenesis. The risk of cancer is correlated with exposure to xenobiotics or endogenous substances, which may be modified in metabolic detoxification activities by genetic variation [4]. It is well known that deletion variants of the GSTM1, GSTT1 and GSTP1 loci could result in loss of functional activity. Hendersonet *et al.* [5] found that GSTP1 null mice, disposed with carcinogen polycyclic aromatic hydrocarbons, had a highly significantly increased risk of skin cancer, and****demonstrated that GSTP1 may be an important determinant in cancer susceptibility. Chromosomal polymorphisms in genes involved in DNA repair, cell cycle control, apoptosis or metabolic enzymes may determine individual susceptibility to cancer [6].

Variants of prevalent allele in every GSTs gene may lower the efficiency of detoxification and aggravate the susceptibility to cancer. It was identified that there are two single nucleotide polymorphisms in GSTP1 that lead to changes in amino acids. One at nucleotide 313 (codon 104) in exon 5 (rs1695) has an A to G transition, which results in an amino acid change from isoleucine (*Ile*) to valine (*Val*). The other has a C to T transition in nucleotide 341 (codon 113) in exon 6 (rs1138272) which results in an amino acid change from alanine (*Ala*) to valine (*Val*) **(Figure S1)**. This two loci were reported to cause minimal catalytic activity in individuals who carry one or more copies of the allele G or T [7], [8].

A large number of studies have investigated the role of GSTP1 polymorphism in the etiology of cancer of various organs, including lung, breast, colorectal, bladder, prostate and so on. However, most of these studies contained small number of subjects, and the results of these studies remain conflicting rather than conclusive. In recent years, plenty of meta-analysis studies were extensively carried out to quantitatively evaluate the association between GSTP1 and cancer susceptibility. As a consequence, statistically significant increased risk was found between GSTP1 313A>G and breast cancer [9], esophageal cancer [10], bladder cancer [11], leukemia [12] and other cancers. Nevertheless, no pooled evaluation on GSTP1 341C>T has been performed yet. In light of the ubiquity of 341C>T polymorphism and the critical role of GSTP1 in the carcinogenic process, we performed a meta-analysis on all eligible case-control studies to estimate the cumulative cancer risk of this polymorphism, and quantitatively analyze the potential influencing factors, with an expectation to provide the most comprehensive evidence for the relationship between this variant and cancer susceptibility.

## Materials and Methods

### Search Strategy

We searched the PubMed, Embase, ISI Web of Knowledge and China National Knowledge Infrastructure for all articles of the association between GSTP1 341C>T polymorphism and cancer risk. The following terms were used in various combinations in this search: ("glutathione S-transferase P1" OR "GSTP1") AND ("polymorphism" OR "variant" OR "variation") AND ("neoplasm" OR "cancer" OR "carcinoma" [last search was updated on August 30, 2012]). All qualified studies were retrieved, and their references were manually checked for additional related publications. Review articles and references of other relevant studies identified were hand-searched as well to search for extra eligible studies. This search strategy was performed iteratively until no further pertinent article was found. Only published studies with full-text articles were included, without language limited. Non-Chinese or non-English articles were translated by Google Translate. When overlapping data on the same subjects were included in more than one publication, only the one with the larger sample size was included in this meta-analysis.

### Inclusion and Exclusion Criteria

Eligible studies in the current meta-analysis had to meet all the following criteria: (a) evaluating the association between GSTP1 Ala114Val polymorphism and cancer risks, (b) using a case-control design, (c) sufficient data of genotypes (CC, CT and TT) were presented with estimated odds ratios (ORs) and 95% confidence intervals (CIs). The major exclusion criteria were: (a) genotype distribution of the control population was deviated from Hardy-Weinberg equilibrium (HWE), (b) family-based control or lack of the control group, (c) duplicated studies.

### Data Extraction

Data were independently extracted in duplicate by two investigators (Shengxin Huang, Feixiang Wu) using a standard protocol and data-collecting form according to the inclusion criteria. Original extraction data were checked by another investigator (Min Luo), and any disagreement was resolved by discussion among the three investigators. The following information was collected from each study: first author's surname, year of publication, country, and ethnicity of the study population, cancer types, and source of control groups, genotyping methods, and frequency of genotypes in both groups. Diverse ethnicity descents were categorized as Asian, African, and Caucasian. If a study was impossible to separate participants according to such phenotypes, the group reported was termed as ''mixed ethnicity''. For studies which included subjects of diverse ethnic groups or cancer types, data were extracted separately if possible.

### Quality Assessment

We developed a quality assessment scale (**Table 1**), which was modified from previous studies [13]–[15], to evaluate the quality of eligible studies. Three reviewers (Min Luo, Liang Ma, Kefeng Gao) independently assessed the quality of studies according to the scale for quality assessment. Any disagreement was resolved by discussion among reviewers. The evaluation mainly focused on representativeness of both cases and controls, specimens of cases for determining genotypes, quality control of genotyping method, sample size. Total score ranged from 0 (worst) to 12 (best). Reports scoring ≥9 (≥75%) were classified as ''high-quality'', scoring 6–8 (50%–75%) as ''moderate-quality'', <50% as "low-quality".

**Table 1:** Scale for quality assessment.

Parameter	Score
**1. Source of cases**	
Selected from population or cancer registry	2
Selected from oncology department or cancer institute	1
No description	0
**2. Representativeness of controls**	
Population-based	2
Population-hospital mixed	1.5
Hospital-based	1
No description	0
**3. Diagnosis of cancer**	
Histological or pathologically confirmed	2
Patient medical record	1
No description	0
**4. Specimens of cases for genotyping**	
Peripheral blood or normal tissues	2
Tumor tissues or exfoliated cells	1
No description	0
**5. Quality control of genotyping**	
Different genotyping assays confirmed the result	2
Quality control by repeated assay	1
No description	0
**6. Total sample size**	
>1000	2
200–1000	1
<200	0

### Statistics Method

Firstly, the allelic frequency was calculated, and the observed genotype frequencies of the GSTP1 341C>T polymorphism were assessed for Hardy-Weinberg equilibrium using χ^2^ test in the control group in each study. The strength of the association between the GSTP1 341C>T polymorphism and cancer risk was assessed by calculating ORs with 95% CIs. The overall pooled analysis was performed for homozygote comparison (TT versus CC), heterozygote comparison (CT versus CC), dominant model (TT/CT versus CC) and the recessive model (TT versus CT/CC), respectively. Comparison of the various subjects, stratified analyses were processed according to ethnicity, source of controls, sample size, matched control, quality score and cancer types (if one cancer type contained less than two individual studies, it would be combined into other cancers group). To achieve sufficient statistical power, we only conducted the meta-analysis on cancer types with more than three studies. Otherwise, we merged the studies into the ''other cancer'' group. The significance of the pooled ORs was determined by the Z-test and *P*<0.05 was considered as statistically significant. We also computed the power of the selected studies, in order to assess the probability of detecting an association between this polymorphism and cancer at the 0.05 level of significance, assuming genotypic risks of 1.2, 1.5 and 2.0.

The heterogeneity of studies was assessed through the Cochran's Q-test and the *I* ^2^ test. If *P*<0.1 or *I* ^2^>50%, it was considered substantial heterogeneity among studies, and the random-effects model (the DerSimonian and Laird method) was applied as the preferred method for estimating the summary ORs and 95% CIs; the fixed-effects model (the Mantel-Haenszel method) was used when there was without substantive heterogeneity. Moreover, sensitivity analysis, by which a single study in the meta-analysis was deleted each time to determine the influence of the individual data set for the overall pooled OR, was performed to assess the stability of the results. To assess the potential publication bias, visual inspection of Begger's funnel plot symmetry [16] was performed. Egger's test was also conducted to analyze the publication bias statistically. The meta-analysis was conducted using Stata software (version 12.0; StataCorp LP, College Station, Texas), with all the *P* values were two-sided. Power was calculated using the power and sample size calculation software PS version 3.0 (http://biostat.mc.vanderbilt.edu/twiki/bin/view/Main/PowerSampleSize). [17].

## Results

### Characteristics of Studies

**Figure 1:**
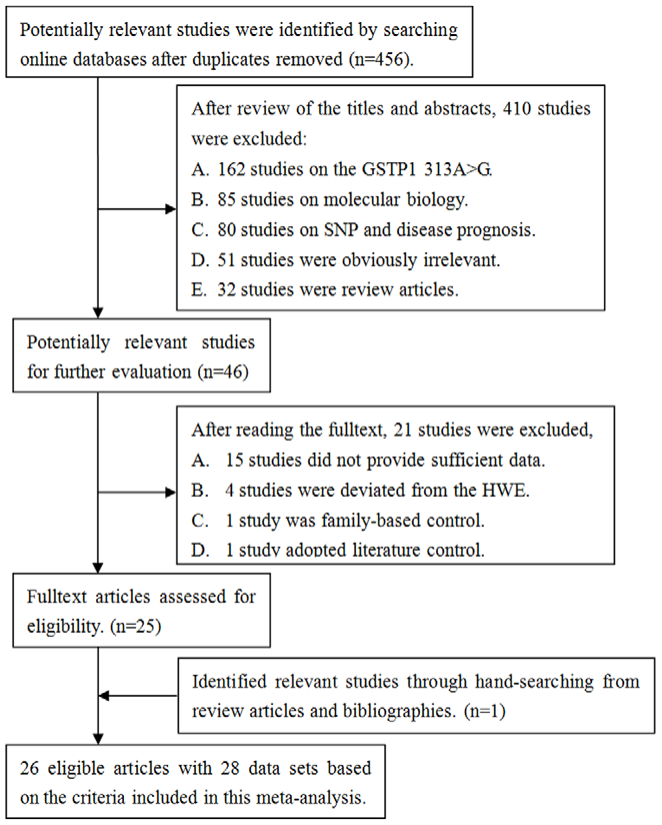
Studies identified with criteria for inclusion and exclusion.

As showed in **Figure 1**, 46 studies exploring the relationship between GSTP1 polymorphisms and cancer susceptibility were identified. After reading the full texts, 21 studies were excluded because they did not provide allele frequencies, which were needed for OR calculation, deviated from the HWE, family-based control or lack of the control group. In addition, one qualified study was identified from review articles and bibliography through hand searching. Finally, a total of 26 eligible publications with 28 case-control studies [18]–[43] met the preset inclusion criteria, in which 13249 cases and 16798 controls were included for the pooled analysis. All studies' characteristics were summarized in **Table 2**. All the studies were published in English except for one [42]. This meta-analysis included seven colorectal cancer studies, six lung cancer studies, four breast cancer studies, three upper digestive tract cancer studies, two thyroid cancer studies, and six other cancer studies (including lymphoma, melanoma *etc.*). There were 14 population-based studies, 13 hospital-based studies and one population-hospital mixed study [20]. Nineteen of 28 studies were conducted in Caucasians; four studies were conducted in Asians [18], [21], [34], [38]; one study was conducted in Africans [36], and four studies of mixed population [23], [30], [40].

**Table 2:** Characteristics of studies included in the meta-analysis.

Author	Publication year	Country	Ethnicity	Cancer type	Control source	Genotyping methods	Matching criteria	n(case)	n(control)	MAF	Power(%)*			Quality score
											1.2	1.5	2	
Al-Dayel	2008	Saudi Arabia	Asian	Lymphoma	PB	PCR-RFLP	Age, sex	145	510	0.126	11.4	36.9	80.5	5
Landi	2005	Spain	Caucasian	Coleractal	HB	APEX	NA	360	325	0.055	8.6	25.6	65.8	5
Barnette	2004	USA	Caucasian	Leukaemia	HB	AS-PCR	NA	170	288	0.075	8.6	24.1	60.1	5
Wadelius	1999	Sweden	Caucasian	Prostate	PB	PCR-SSCP	Age	171	148	0.095	7.7	20.4	53.1	6
Van Emburgh	2008	USA	African	Breast	HB	PCR-SSCP	Age, ethnicity	54	74	0.027	5.7	7.6	12.9	6
Ibarrola-Villava	2012	Spain	Caucasian	Melanoma	HB	TaqMan	Region	555	359	0.025	6.8	16.1	43.5	6
Ebrahimkhani	2012	Iran	Asian	Coleractal	HB	Pyrosequencing	NA	73	95	0.063	6.3	11.2	25.1	6
Marciniak	2006	Poland	Caucasian	Thyroid	HB	PCR-RFLP	NA	103	53	0.113	5.9	11.2	27.3	6
Siraj	2008	Saudi Arabia	Asian	Thyroid	PB	PCR-RFLP	Age	40	510	0.126	8.2	18.8	43.3	7
Van Emburgh	2008	USA	Caucasian	Breast	HB	PCR-SSCP	Age, ethnicity	386	385	0.064	9.8	32.1	77.3	7
Saarikoski	1998	Finland	Caucasian	Lung	PB	PCR-RFLP	NA	206	293	0.090	9.5	29.2	70.9	8
Jiao	2007	USA	Caucasian	Pancreatic	HB	MassCode PCR	Age, sex	335	298	0.096	10.6	36.6	82.7	8
Kim	2008	Germany	Caucasian	Breast	HB	MALDI-TOF MS	Age	596	609	0.076	14.0	52.5	95.4	8
Zienolddiny	2008	Norway	Caucasian	Lung	PB	APEX	Age, sex, smoking	319	381	0.066	9.7	30.8	74.4	8
Wang	2011	India	Asian	Coleractal	PB	PCR-RFLP	Age, sex	302	291	0.050	7.9	21.4	56.2	8
Murphy	2007	Ireland	Caucasian	Oesophageal	PB	Multiplex PCR	Age, sex	207	223	0.078	8.3	23.3	59.8	9
Küry	2008	France	Caucasian	Coleractal	PB	TaqMan	Age, sex	1023	1121	0.073	20.8	75.7	99.8	9
García-Closas	2005	Spain	Caucasian	Bladder	HB	TaqMan	Age, sex, ethnicity, region	1083	1007	0.047	14.9	56.9	97.2	9
MARIE-GENICA	2010	Germany	Caucasian	Breast	PB	MALDI-TOF MS	Age, region	3148	5487	0.086	66.5	100.0	100.0	9
sorensen	2004	Danmark	Caucasian	Lung	PB	PCR-RFLP	NA	253	266	0.086	9.3	28.9	71.3	9
Northwood	2010	UK	Caucasian	Coleractal	PB	TaqMan	NA	308	296	0.111	11.2	38.9	85.5	9
Yang	2004	USA	Mixed	Lung	PB	TaqMan	NA	229	229	0.092	9.0	27.3	68.3	9
Landi	2007	Italy	Caucasian	MPM	PB	APEX	NA	88	391	0.051	7.8	17.1	39.9	9
Canova	2009	Italy	Caucasian	UDTC	Miexed	APEX	Age, sex, ethnicity, region	1501	1449	0.073	26.7	87.5	100.0	9.5
wang	2003	USA	Caucasian	Lung	PB	PCR-RFLP	Age, sex, ethnicity, smoking	579	598	0.075	13.7	51.1	94.8	10
Moore	2005	USA	Mixed	Coleractal	HB	TaqMan	Sex, ethnicity	700	714	0.085	16.8	63.4	98.6	10
Harris	1998	Australia	Mixed	Coleractal	HB	PCR-RFLP	NA	131	199	0.073	7.6	18.7	47.2	10
Harris	1998	Australia	Mixed	Lung	HB	PCR-RFLP	NA	184	199	0.073	7.8	20.3	52.6	10

MPM, malignant pleural mesothelioma; UDTC, upper digestive tract cancer; HB, hospital–based control; PB, population-based control; APEX, arrayed primer extension; PCR, polymerase chain reaction; RFLP, restriction fragment length polymorphism; SSCP, single strand conformation polymorphism; AS, allele-specific; ASO, allele-specific oligonucleotide; MALDI-TOF-MS, matrix-assisted laser desorption/ionization time-of-flight-mass spectrometry; NA: not available.

* Power was calculated by the PS software with MAF in controls as the frequency of risk factor and OR was selected with 1.2, 1.5 and 2 as the relative risk.

Generally, the quality of these included studies was satisfactory. Cancers were confirmed by histology or pathology in 78.6% (22/28) studies. Most of the studies described genotyping measures of controls, such as positive and/or negative controls (4/28), blindness to case-control status (5/28), confirmation by different genotyping methods(2/28) and random repetition in 50%(14/28) studies. 60.7% (17/28) studies used matched control population by age, gender, ethnicity or other factors. Most of the included studies adopted rational sample size, and 60.7% (17/28) of all studies were with large sample size (>500). The total sample size of each group was more than 1000 in seven studies. However, only two studies had statistical power over 80% when we assumed an allelic OR of 1.5. According to the preset Quality Assessment Scale, most of the included studies were identified as ''moderate-quality'' (13 studies) and ''high-quality'' (12 studies), only three studies as "low-quality". Different genotyping methods were utilized in these studies, including the classical method PCR-RFLP, TaqMan assay, APEX technology, MALDI-TOF MS and other methods in the remaining studies.

### Quantitative Synthesis

**Figure 2:**
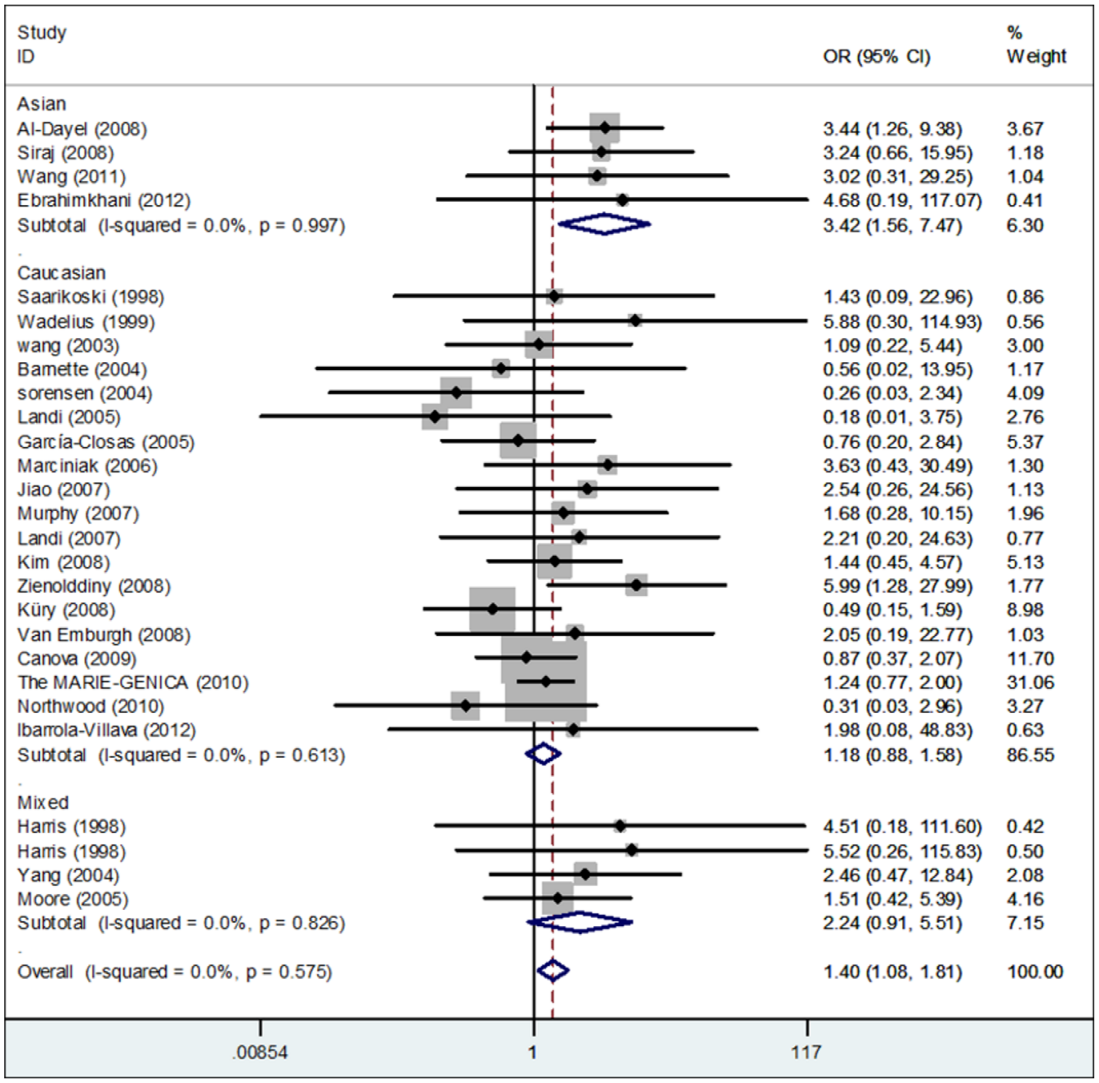
Forest plot of cancer risk associates with GSTP1 341C>T polymorphism under homozygote comparison (TT versus CC) in different ethnicity. African ethnic is omitted due to the insufficient data. The squares and horizontal lines correspond to the study-specific OR and 95% CI. The area of the squares reflects the study specific weight. The diamond represents the pooled OR and 95% CI.

**Table 3** lists the main results of this pooled analysis and **Figure 2** **and** **3** show the association of GSTP1 341C>T polymorphism with cancer risk in the form of forest plots. For the overall analysis, significant association between the risk of cancer and the variant genotypes of GSTP1 341C>T polymorphism was found in homozygote comparison (TT versus CC: *P* = 0.012, OR = 1.40, 95% CI: 1.08–1.81, *P*_het._ = 0.575), and recessive model (TT versus CT/CC: *P* = 0.012, OR = 1.40, 95% CI: 1.08–1.81, *P*_het._ = 0.562). However, no significant association was found in heterozygote comparison (CT versus CC) nor the dominant model (TT/CT versus CC).

**Figure 3:**
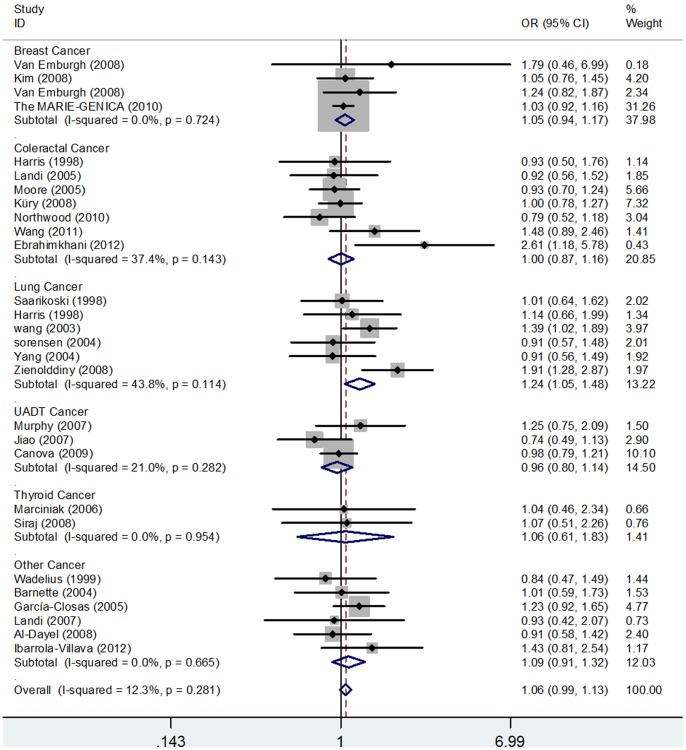
Forest plot of cancer risk associate with GSTP1 341C>T polymorphism under dominant model (TT/CT versus CC) of different types of cancers. The squares and horizontal lines correspond to the study-specific OR and 95% CI. The area of the squares reflects the study specific weight. The diamond represents the pooled OR and 95% CI.

**Table 3:** Stratified analyses of the GSTP1 341C>T polymorphism on cancer risk.

Variables	N	TT versus CC			CT versus CC			TT/CT versus CC (Dominant model)			TT versus CT/CC (Recessive model)		
		OR (95% CI)	Phet.	I2(%)	OR (95% CI)	Phet.	I2(%)	OR (95% CI)	Phet.	I2(%)	OR (95% CI)	Phet.	I2(%)
**Overall**	28	**1.40(1.08–1.81)**	0.575	0.0	1.04(0.97–1.11)	0.305	10.6	1.06(0.99–1.13)	0.281	12.3	**1.40(1.08–1.81)**	0.562	0.0
**Cancer types**													
Colorectal cancer	7	0.91(0.48–1.71)	0.349	10.5	1.01(0.87–1.17)	0.220	27.3	1.00(0.87–1.16)	0.143	37.4	0.90(0.48–1.71)	0.375	7.0
Lung cancer	6	1.96(0.97–3.92)	0.274	21.2	**1.21(1.02–1.45)**	0.197	31.8	**1.25(1.05–1.48)**	0.114	43.8	1.90(0.95–3.82)	0.292	18.7
UDTC	3	1.11(0.54–2.28)	0.605	0.0	0.95(0.79–1.14)	0.245	28.8	0.96(0.80–1.14)	0.282	21.0	1.11(0.54–2.29)	0.593	0.0
Breast cancer	4	1.29(0.84–1.99)	0.902	0.0	1.04(0.93–1.16)	0.730	0.0	1.05(0.94–1.17)	0.724	0.0	1.28(0.83–1.98)	0.908	0.0
Thyroid cancer	2	3.45(0.89–13.29)	0.929	0.0	0.85(0.47–1.55)	0.786	0.0	1.06(0.61–1.83)	0.954	0.0	3.56(0.93–13.69)	0.913	0.0
Other cancer	6	1.94(0.98–3.83)	0.505	0.0	1.05(0.87–1.28)	0.306	16.7	1.10(0.91–1.32)	0.665	0.0	**1.98(1.01–3.91)**	0.447	0.0
**Ethnicity**													
African	1	NC			1.48 (0.33–6.65)^a^			1.48 (0.33–6.65)^a^			NC		
Asian	4	**3.42(1.56–7.47)**	0.997	0.0	1.19(0.72–1.98)^b^	0.052	61.2	1.24(0.94–1.64)	0.120	48.6	**3.49(1.59–7.63)**	0.998	0.0
Caucasian	19	1.18(0.88–1.58)	0.613	0.0	1.05(0.98–1.13)	0.349	8.7	1.06(0.99–1.14)	0.241	17.5	1.17(0.88–1.57)	0.631	0.0
Mixed	4	2.24(0.91–5.51)	0.826	0.0	0.91(0.74–1.13)	0.932	0.0	0.96(0.77–1.18)	0.926	0.0	2.28(0.93–5.60)	0.828	0.0
**Source of controls**													
Population-based	14	**1.45(1.05–1.99)**	0.206	22.9	1.04(0.96–1.14)	0.203	23.1	1.06(0.98–1.16)	0.163	27.2	**1.45(1.05–1.99)**	0.196	23.8
Hospital-based	13	1.52(0.89–2.58)	0.882	0.0	1.06(0.93–1.20)	0.373	7.3	1.08(0.95–1.22)	0.422	2.5	1.52(0.89–2.57)	0.879	0.0
**Matched control**													
Yes	17	**1.42(1.07–1.89)**	0.468	0.0	1.06(0.98–1.14)	0.133	28.3	1.07(0.99–1.15)	0.137	27.9	**1.42(1.07–1.89)**	0.444	0.7
No	11	1.28(0.68–2.42)	0.510	0.0	0.97(0.82–1.14)	0.728	0.0	0.98(0.83–1.16)	0.669	0.0	1.29(0.68–2.44)	0.518	0.0
**Quality score**													
≥9	12	1.08(0.79–1.48)	0.700	0.0	1.03(0.95–1.11)	0.670	0.0	1.03(0.96–1.11)	0.699	0.0	1.078(0.79–1.48)	0.697	0.0
6–8	13	**2.82(1.56–5.09)**	0.982	0.0	1.14(0.93–1.39)^b^	0.093	37.3	1.21(0.99–1.47)^b^	0.080	39.1	**2.78(1.54–5.03)**	0.986	0.0
≤5	3	1.09(0.15–7.81)^b^	0.116	53.5	0.89(0.67–1.19)	0.588	0.0	0.94(0.71–1.25)	0.954	0.0	1.10(0.14–8.36)^b^	0.103	56.0
>5	25	**1.36(1.04–1.79)**	0.715	0.0	1.05(0.98–1.13)	0.257	14.5	1.07(0.99–1.14)	0.186	19.9	**1.36(1.03–1.78)**	0.729	0.0

UDTC, upper digestive tract cancer; Phet., *P* value of Q -test for heterogeneity test; NC, no insufficient data for calculation; N, number of studies for comparison; The figures given in bold indicate statistically significant values.

a OR adjusted for age, family history, smoking history, age of menarche, age of first birth and body mass index according to the author.

b Random-effect model was used.

In a stratified analysis by cancer type, significant risk was observed in lung cancers in heterozygote comparison (CT versus CC: *P* = 0.033, OR = 1.21, 95% CI: 1.02–1.45, *P*_het._ = 0.197) and dominant model (TT/CT versus CC: *P* = 0.013, OR = 1.25, 95% CI: 1.05–1.48, *P*_het._ = 0.114). Interestingly, statistically significantly ascending cancer risk was also observed in ''other cancers'' in the recessive model (TT versus CT/CC: *P* = 0.048, OR = 1.98, 95% CI: 1.01–3.91, *P*_het._ = 0.447). No significant association was found between this polymorphism and thyroid cancer, breast cancer, upper digestive tract cancers and colorectal cancer. In subgroup analysis according to ethnicity, significantly increased risk was found in the Asian population (TT versus CC: *P* = 0.002, OR = 3.42, 95% CI: 1.56–7.47, *P*_het._ = 0.997; TT versus CT/CC: *P* = 0.002, OR = 3.49, 95% CI: 1.59–7.63, *P*_het._ = 0.998), but this association was not found in the African, Caucasian and mixed population. After stratified analysis by the source of controls, significantly increased risk was observed in population-based studies (TT versus CC: *P* = 0.023, OR = 1.45, 95% CI: 1.05–1.99, *P*_het._ = 0.206; TT versus CT/CC: *P* = 0.024, OR = 1.45, 95% CI: 1.05–1.99, *P*_het._ = 0.196). Moreover, this association was found in those studies with matched controls (TT versus CC: *P* = 0.015, OR = 1.42, 95% CI: 1.07–1.89, *P*_het._ = 0.468; TT versus CT/CC: *P* = 0.016, OR = 1.45, 95% CI: 1.07–1.89, *P*_het_ = 0.444). According to the quality assessment, this association was found in those ''moderate-quality'' studies (TT versus CC: *P* = 0.001, OR = 2.82, 95% CI: **1.56–5.09**, *P*_het._ = 0.982; TT versus CT/CC: *P* = 0.001, OR = 2.78, 95% CI: 1.54–5.03, *P*_het._ = 0.986), and this similar result was also detected when three ''low-quality'' studies were excluded (TT versus CC: *P* = 0.023, OR = 1.36, 95% CI: 1.04–1.79, *P*_het._ = 0.715; TT versus CT/CC: *P* = 0.029, OR = 1.36, 95% CI: 1.03–1.78, *P*_het._ = 0.729).

### Heterogeneity and Sensitivity Analysis

For the overall comparisons, no significant heterogeneity among studies was observed (the four genetic model comparison all *P*_het._>0.1), which suggested that there was no substantial heterogeneity between studies, except significant heterogeneity in the stratified analysis according to ethnicity (CT versus CC: *P*_het._ = 0.052, *I* ^2^ = 61.2%), for which the random-effect model was conducted in the pooled analysis. In the sensitivity analyses, the influence of each study on the pooled OR was checked by repeating the meta-analysis while omitting each study, one at a time. The corresponding pooled ORs were not materially altered, indicating that our results were statistically robust (**Table S1**).

### Publication Bias

**Figure 4:**
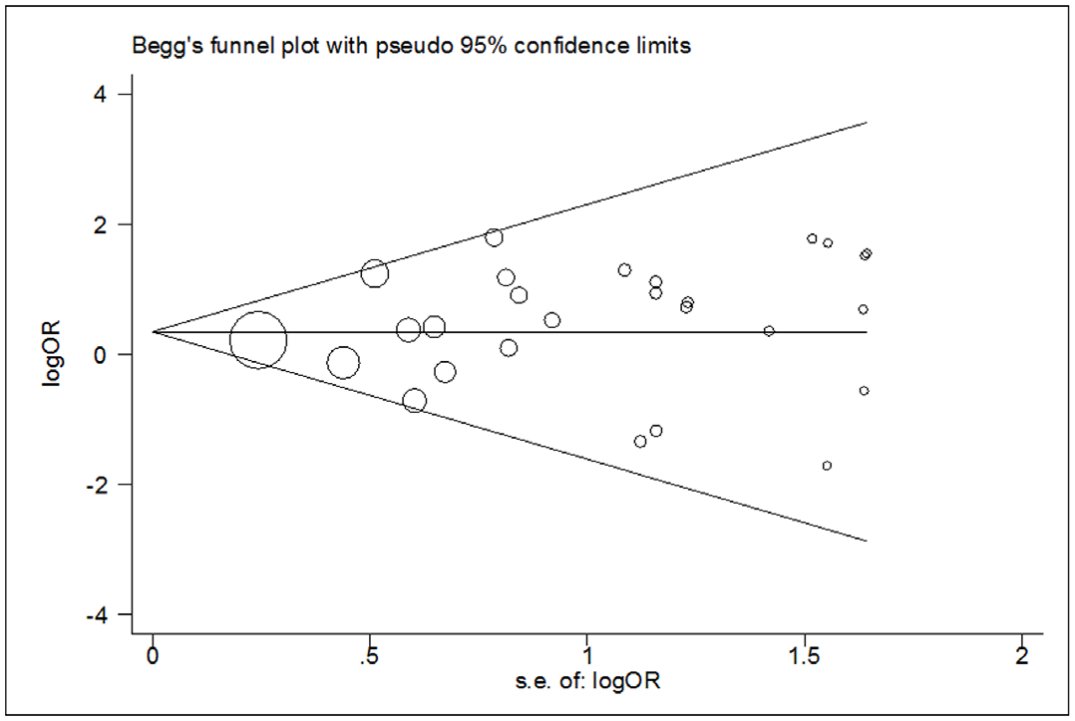
Begg's funnel plot for publication bias test (TT versus CC). Each point represents a separate study for the indicated association. LogOR, natural logarithm of OR. Horizontal line, mean effect size.

Begger's funnel plot and Egger's test were performed to assess the publication bias of articles. As it showed in **Figure 4** **and** **5**, the shapes of the funnel plots did not reveal any evidence of an obvious asymmetry in all comparison models. Moreover, Egger's test was used to provide statistical evidence of funnel plot symmetry. The results still did not show any evidence of publication bias (*t* = 0.99, *P* = 0.330 for TT versus CC; *t* = 0.41, *P* = 0.688 for CT versus CC; *t* = 0.99, *P* = 0.332 for dominant model; *t* = 0.96, *P* = 0.346 for recessive model).

**Figure 5:**
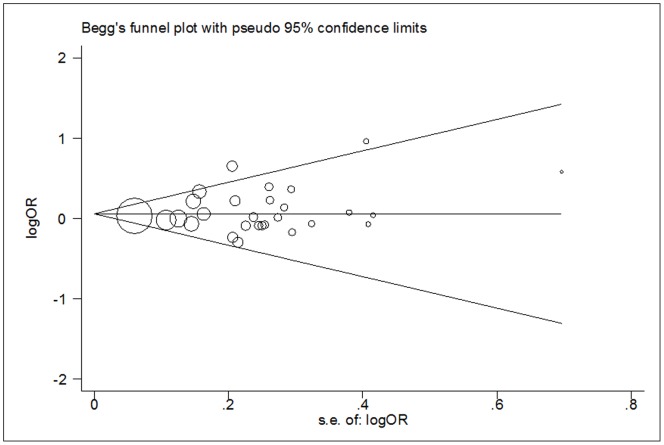
Begg's funnel plot for publication bias test (TT/CT versus CC). Each point represents a separate study for the indicated association. LogOR, natural logarithm of OR. Horizontal line, mean effect size.

## Discussion

As detoxifying enzyme, glutathione S-transferase plays an important role in protecting cells from cytotoxic and carcinogenic agents in the defense system. The GSTP1 gene is located on chromosome 11q13, and previous studies have indicated that it was widely expressed but predominantly in normal epithelial cells, such as the respiratory tract system, digestive system, and urinary system [44]. Exon 5 (313A>G) and exon 6 polymorphisms (341C>T) were reported that they could result in decreased conjugating activity [8]. The GSTP1 313A>G polymorphism was shown to be a predisposing risk factor for a number of human malignancies. Accordingly, we speculated that GSTP1 341C>T polymorphism might also be a risk factor in the genesis and development of cancer. Previous conclusions of numerous studies on the association between the GSTP1 341C>T polymorphism and cancer risk remain conflicting and contradictory. The inconsistent results are possibly because of a slight effect of the polymorphism on cancer risk or the relatively low statistical power of published studies. Consequently, meta-analysis is needed to provide a quantitative approach for combining the different results.

The present meta-analysis, which included 13249 cases and 16798 controls from 26 publications with 28 case-control studies, explored the relationship between the 341C>T polymorphism and cancer risk. For overall comparison of pooled ORs, significantly increased risk was observed in homozygote comparison (TT versus CC) and the recessive model (TT versus CT/CC), without significant between-study heterogeneity. These results indicated that GSTP1 341C>T polymorphism may increase cancer risk, especially in individuals with mutant allele homozygous TT genotype.

Previous studies have proved that GSTP1 was variably expressed in malignant tumor tissues with a low level in lymphoma and breast cancer, while over-expressed were found in lung cancer, head and neck tumors and colon cancer [45]. GSTP1 exert varying effects on different organs, and the carcinogenic mechanism difference may account for varied cancer risk. Our meta-analysis provide evidence that the variant GSTP1 341C>T may enhance individual susceptibility to lung cancer when special cancer was concerted. This result demonstrates the disparate distribution of GSTP1 in different sites, and the over-expressed GSTP1 may be a risk of cancer. Furthermore, genetic variability in the individual response to carcinogens might modify the susceptibility to cancer [46]. Van Emburgh *et.al* [36]found that the effect of the GSTP1 SNPs appeared to be modulated by smoking history in a case-control study, leading to a 2-fold increase in breast cancer risk, which was also observed in smokers with the 341T allele. Meanwhile, it's worth noting that statistically increased risk was observed in ''other cancer'', which was combined with a number of single studies with different cancer types, implying that significant association may really exist. But due to the low statistical power, further well-designed studies with larger sample size based on specific cancer need to be carried out to re-examine this correlation.

In the stratified analysis based on ethnicity, no significant risk of cancer was found except for the Asian population. We simply presented the ORs of the one study of African population according to the original result and adjusted by some factor, such as the age, and smoking history [36]. A total of 19 studies(11391 cases and 13977 controls), which were determined to be adequate for supporting the observed result, investigated the relationship between this polymorphism and cancer risk among Caucasian, but no significant risk was found. The pooled analysis of the Asian population, including four single studies with 1966 subjects, reached a 78% statistical power when we assumed an allelic OR of 1.5. A significantly elevated risk of cancer was discovered in the homozygote comparison and recessive model of the Asian population; the pooled ORs are 3.42- and 3.49-fold of the referenced genotype. Our results demonstrated that there was a moderate association between the GSTP1 341C>T polymorphism and cancer risk in Asian population. Although the reason for these discrepancies is not positively known, some possibilities should be considered, such as the differences in the underlying environment-gene interaction and lifestyle. Nevertheless, additional larger sample size study or genome-wide association study is warranted for further validation of this finding.

When stratifying the source of controls, a weak strength was observed in population-based control studies (OR = 1.45, 95% CI: 1.05–1.99 for both homozygote comparison and recessive model), but not in the hospital-based control studies. After attentive observation of the strength of association, it was found that not only the ORs were closed to the overall comparison, but also in the same genetic model. The hospital-based control studies may have an inherent selection bias since the controls may not be representative of the general population, particularly as the controls may be associated with the disease-related conditions, such as benign inflammatory lesions, which were the confounding factors for investigation. Analogously, evidently increased cancer risk was detected in matched control studies, but not in those studies without matched control. The matching criteria of these studies were mostly matched with age, sex and ethnic. In addition, some matched with region and lifestyle, for example, smoking or alcohol consumption. Apparently, matching criteria play an important role in genetic polymorphism and disease association studies, as it may avoid selection bias for control and reduce the impact of potential confounding factors on the investigation. All the studies were divided into three levels in accordance with the quality assessment scale. Significantly increased cancer risk was detected in the ''moderate-quality'' studies, but not the ''high-quality'' studies. There were three Asian population studies in this ''moderate-quality'' level, which may account for this phenomenon. It was noteworthy that the pooled ORs were not materially changed from the result of overall comparison, when removed three ''low-quality'' studies. It's indicated that the overall comparison results were statistically robust when excluding the low-quality studies.

To the best of our knowledge, this is the first and the most comprehensive meta-analysis undertaken so far for quantitative analyses between GSTP1 341C>T polymorphism and the risk of cancer. However, in interpreting the outcome of this meta-analysis, some limitations need to be addressed. Firstly, there was insufficient data on the African population in this meta-analysis. Secondly, this meta-analysis was based on unadjusted evaluation, and original data shortage limited our further evaluation of potential gene-environment interactions. In order to provide a more precise estimation based on adjustment of confounding factors, well-designed studies are warranted by taking potential confounding factors such as smoking and alcohol consumed into account. In spite of these, our meta-analysis still has some advantages. On one hand, all the studies included in the current meta-analysis met our selection criteria, and all the pooled ORs based on no substantial heterogeneity between studies. On the other hand, there was not any publication bias detected, which indicates that the entire pooled result is robust and authentic. Besides, the subject number of more than 30 000 in the published studies is sufficient for a comprehensive analysis, which dramatically increases the statistical power of the analysis.

In conclusion, our meta-analysis suggests that the GSTP1 341C>T polymorphism may implicate genetic susceptibility to cancer. Since only one study from the African population and four studies from the Asian population, it is critical that a larger sample size and well-designed multi-center studies based on these two ethnic groups should be performed to further re-evaluate the association. Furthermore, investigation of the impact of gene-gene and gene-environment interactions on the GSTP1 341C>T polymorphism and cancer risk is necessary for providing a better comprehensive understanding of the association.

## Supporting Information

Figure S1 **Genomic structure of human GSTP1 gene and location of the 341C>T polymorphism.**
(TIF)

Table S1 **ORs (95% CI) of sensitivity analysis for the meta-analysis.**
(DOC)

File S1 **MOOSE Checklist for the meta-analysis.**
(DOC)
