# Supplementary figures and images for: The Glutathione S-Transferase P1 341C>T Polymorphism and Cancer Risk: A Meta-Analysis of 28 Case-Control Studies
Source: PLoS One. 2013 Feb 21;8(2):e56722. doi: 10.1371/journal.pone.0056722 (PMC3578943; doi:10.1371/journal.pone.0056722)

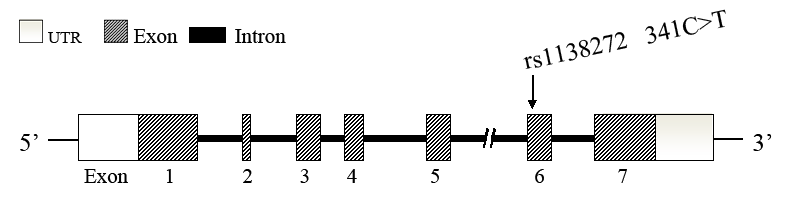

Supplement: Figure S1 — Genomic structure of human GSTP1 gene and location of the 341C>T polymorphism. (TIF) [file pone.0056722.s001.tif]
