# Supplementary material for: The Glutathione S-Transferase P1 341C>T Polymorphism and Cancer Risk: A Meta-Analysis of 28 Case-Control Studies
Source: PLoS One. 2013 Feb 21;8(2):e56722. doi: 10.1371/journal.pone.0056722 (PMC3578943; doi:10.1371/journal.pone.0056722)
Supplement: Table S1 — ORs (95% CI) of sensitivity analysis for the meta-analysis. (DOC) [file pone.0056722.s002.doc]

Table S1. ORs (95% CI) of sensitivity analysis for overall comparison

1. **TT versus CC**

----------------------------------------------------------------------------

Omitted study | Estimate [95% Conf. Interval]

-------------------------+--------------------------------------------------

Van Emburgh (2008) | 1.3970524 1.0769308 1.8123315

Siraj (2008) | 1.3750736 1.0569712 1.7889111

Al-Dayel (2008) | 1.3190559 1.0074853 1.7269814

Wang (2011) | 1.3799581 1.0615188 1.7939244

Ebrahimkhani (2012) | 1.3836916 1.0653994 1.797075

Wadelius (1999) | 1.3716593 1.055412 1.782668

wang (2003) | 1.406491 1.0804177 1.8309741

Saarikoski (1998) | 1.3968 1.0755002 1.8140865

Van Emburgh (2008) | 1.390183 1.0698864 1.8063682

Jiao (2007) | 1.3839896 1.0647216 1.7989934

Murphy (2007) | 1.3914647 1.0696192 1.8101525

Küry (2008) | 1.4868351 1.1366387 1.9449264

Kim (2008) | 1.3946556 1.0676982 1.821736

García-Closas (2005) | 1.4332586 1.0988232 1.8694819

Zienolddiny (2008) | 1.3141406 1.0066103 1.7156247

Canova (2009) | 1.4662717 1.1156237 1.9271307

The MARIE-GENICA (2010) | 1.4683157 1.075518 2.0045701

Ibarrola-Villava (2012) | 1.393322 1.0730483 1.8091882

Landi (2005) | 1.4316538 1.1013551 1.8610098

sorensen (2004) | 1.4455297 1.110706 1.8812864

Northwood (2010) | 1.433902 1.1024594 1.8649892

Barnette (2004) | 1.406871 1.0832998 1.8270899

Marciniak (2006) | 1.3676144 1.0512071 1.7792584

Landi (2007) | 1.3907537 1.0705706 1.8066962

Moore (2005) | 1.3920372 1.0669884 1.8161092

Harris (1998) | 1.3840113 1.0656477 1.7974863

Harris (1998) | 1.3765239 1.0594428 1.7885043

Yang (2004) | 1.3744197 1.0555557 1.7896068

-------------------------+--------------------------------------------------

Combined | 1.3970524 1.0769308 1.8123315

----------------------------------------------------------------------------

1. **CT versus CC**

------------------------------------------------------------------------------

Omitted study | Estimate [95% Conf. Interval]

------------------------+-----------------------------------------------------

Van Emburgh (2008) | 1.0404484 .97301426 1.1125559

Siraj (2008) | 1.0427416 .97500946 1.1151791

Al-Dayel (2008) | 1.049387 .9807842 1.1227884

Wang (2011) | 1.0363641 .96871904 1.1087328

Ebrahimkhani (2012) | 1.0352532 .96798066 1.1072011

Wadelius (1999) | 1.0462938 .97814009 1.1191963

wang (2003) | 1.0267202 .95867849 1.0995912

Saarikoski (1998) | 1.0425511 .97439032 1.1154798

Van Emburgh (2008) | 1.0373387 .96932366 1.1101263

Jiao (2007) | 1.0520719 .98314074 1.125836

Murphy (2007) | 1.0390485 .97125566 1.1115733

Küry (2008) | 1.0429014 .97292959 1.1179054

Kim (2008) | 1.0423379 .97347727 1.1160696

García-Closas (2005) | 1.0309446 .96245493 1.1043081

Zienolddiny (2008) | 1.0276916 .96027973 1.0998359

Canova (2009) | 1.0483719 .97709387 1.1248497

The MARIE-GENICA (2010)| 1.0517854 .9703822 1.1400173

Ibarrola-Villava (2012)| 1.0374722 .96984571 1.1098142

Landi (2005) | 1.0429631 .97486903 1.1158136

sorensen (2004) | 1.0429598 .97483552 1.1158448

Northwood (2010) | 1.0490348 .9802352 1.1226633

Barnette (2004) | 1.0419237 .97396579 1.1146234

Marciniak (2006) | 1.0435418 .97580568 1.1159798

Landi (2007) | 1.0431434 .97540068 1.115591

Moore (2005) | 1.0497335 .97995831 1.1244769

Harris (1998) | 1.0437293 .97580546 1.1163811

Harris (1998) | 1.041468 .97358482 1.1140844

Yang (2004) | 1.0459928 .97771322 1.1190408

------------------------+-----------------------------------------------------

Combined | 1.0418084 .97436844 1.1139161

------------------------------------------------------------------------------

1. **TT/CT versus CC (Dominant model)**

------------------------------------------------------------------------------

Omitted study | Estimate [95% Conf. Interval]

--------------------------+---------------------------------------------------

Van Emburgh (2008) | 1.0565986 .98962775 1.1281015

Siraj (2008) | 1.0577747 .9905562 1.1295547

Al-Dayel (2008) | 1.0614986 .99355701 1.1340862

Wang (2011) | 1.0518894 .98473781 1.1236203

Ebrahimkhani (2012) | 1.0511078 .98431194 1.1224365

Wadelius (1999) | 1.0610757 .99347991 1.1332706

wang (2003) | 1.0440243 .9763934 1.1163397

Saarikoski (1998) | 1.0587706 .99108869 1.1310746

Van Emburgh (2008) | 1.0534743 .98593925 1.1256354

Jiao (2007) | 1.0673727 .99898735 1.1404393

Murphy (2007) | 1.0549125 .98758932 1.126825

Küry (2008) | 1.0627397 .99302507 1.1373485

Kim (2008) | 1.0580824 .98969676 1.1311933

García-Closas (2005) | 1.049049 .98090281 1.1219296

Zienolddiny (2008) | 1.0406867 .97388455 1.1120711

Canova (2009) | 1.0669991 .99604699 1.1430054

The MARIE-GENICA (2010) | 1.070628 .98959811 1.1582928

Ibarrola-Villava (2012) | 1.0534677 .98632487 1.1251812

Landi (2005) | 1.0604474 .99274513 1.1327668

sorensen (2004) | 1.060833 .99305988 1.1332315

Northwood (2010) | 1.066395 .9980092 1.1394667

Barnette (2004) | 1.0586237 .99111277 1.1307333

Marciniak (2006) | 1.0580171 .99082465 1.1297662

Landi (2007) | 1.0588254 .99156651 1.1306466

Moore (2005) | 1.0654579 .99620727 1.1395224

Harris (1998) | 1.0593099 .99189499 1.1313068

Harris (1998) | 1.0567394 .98938202 1.1286825

Yang (2004) | 1.06077 .99302742 1.1331339

--------------------------+---------------------------------------------------

Combined | 1.0578796 .99090549 1.1293805

------------------------------------------------------------------------------

1. **TT versus CT/CC (Recessive model)**

------------------------------------------------------------------------------

Omitted study | Estimate [95% Conf. Interval]

--------------------------+---------------------------------------------------

Van Emburgh (2008) | 1.3954658 1.0758704 1.8099996

Siraj (2008) | 1.3730622 1.0555846 1.7860243

Al-Dayel (2008) | 1.3128151 1.0028307 1.7186186

Wang (2011) | 1.3792559 1.0611637 1.7926988

Ebrahimkhani (2012) | 1.3839642 1.0658188 1.7970755

Wadelius (1999) | 1.3690012 1.0534839 1.7790155

wang (2003) | 1.4070005 1.0809723 1.8313607

Saarikoski (1998) | 1.3952141 1.0744425 1.8117509

Van Emburgh (2008) | 1.3890742 1.0692045 1.804638

Jiao (2007) | 1.3811143 1.0626396 1.7950362

Murphy (2007) | 1.3907979 1.0692758 1.8089987

Küry (2008) | 1.4853512 1.1356736 1.9426957

Kim (2008) | 1.393291 1.0668178 1.8196732

García-Closas (2005) | 1.4328788 1.0986961 1.8687075

Zienolddiny (2008) | 1.3176206 1.0096074 1.7196032

Canova (2009) | 1.4640523 1.1141114 1.9239092

The MARIE-GENICA (2010) | 1.4679146 1.0753574 2.0037742

Ibarrola-Villava (2012) | 1.3919496 1.07216 1.8071216

Landi (2005) | 1.4299249 1.1001945 1.858476

sorensen (2004) | 1.4437645 1.1095186 1.8787031

Northwood (2010) | 1.4311158 1.1005088 1.8610414

Barnette (2004) | 1.4053141 1.082264 1.8247928

Marciniak (2006) | 1.3640848 1.0485683 1.7745407

Landi (2007) | 1.3890055 1.069392 1.8041432

Moore (2005) | 1.3894819 1.065185 1.8125115

Harris (1998) | 1.3822383 1.0644397 1.7949187

Harris (1998) | 1.3750995 1.0585133 1.7863722

Yang (2004) | 1.3715918 1.053514 1.7857041

--------------------------+---------------------------------------------------

Combined | 1.3954658 1.0758704 1.8099996

------------------------------------------------------------------------------
