# Supplementary material for: The Glutathione S-Transferase P1 341C>T Polymorphism and Cancer Risk: A Meta-Analysis of 28 Case-Control Studies
Source: PLoS One. 2013 Feb 21;8(2):e56722. doi: 10.1371/journal.pone.0056722 (PMC3578943; doi:10.1371/journal.pone.0056722)
Supplement: File S1 — MOOSE Checklist for the meta-analysis. (DOC) [file pone.0056722.s003.doc]

**MOOSE Checklist**

**Article details:** The Glutathione S-Transferase P1 341C>T Polymorphism and Cancer Risk: A Meta-Analysis of 28 Case-Control Studies

**Authors:** Sheng-xin Huang, Fei-xiang Wu, MD, Min Luo, Liang Ma, Ke-feng Gao, Jian Li, Wen-juan Wu, Shan Huang, Qi Yang, Ke Liu, Yin-nong Zhao, Le-qun Li, MD

| **Criteria** | | **Brief description of how the criteria were handled in the meta-analysis** |
| --- | --- | --- |
| **Reporting of background should include** | |  |
| √ | Problem definition | GSTP1, which is one major group of the glutathione S-transferase family, plays an important role in metabolites of carcinogens and toxins, reducing damage of DNA as a suppressor of carcinogenesis. The 341C>T polymorphism of the GSTP1 has been implicated in cancer risk through cutting down its metabolic detoxification activities. However, results from studies remain conflicting rather than conclusive. |
| √ | Hypothesis statement | *GSTP1* 341C>T polymorphism might influence the susceptibility of cancers, which might relate to the ethnicity, cancer type or other aspects. |
| √ | Description of study outcomes | Cancer. |
| √ | Type of exposure or intervention used | TT, CT, **TT/CT** genotypes in *GSTP1* 341C>T. |
| √ | Type of study design used | We included case-control studies. |
| √ | Study population | We placed no restriction. |
| **Reporting of search strategy should include** | |  |
| √ | Qualifications of searchers | The credentials of the two investigators Fei-xiang Wu and Le-qun Li are indicated in the author list. |
| √ | Search strategy, including time period included in the synthesis and keywords | PubMed from 1965 –August 30, 2012;  EMBASE from 1974 –August 30, 2012;  ISI Web of Knowledge from 1950 –August 30, 2012;  China National Knowledge Infrastructure from 1915 –August 30, 2012.  **Retrieving query formulation**: （“glutathione S-transferase P1” OR “GSTP1”） AND (“polymorphism” OR “variant” OR “variation”) AND (“neoplasm” OR “cancer” OR “carcinoma”). |
| √ | Databases and registries searched | PubMed, EMBASE, ISI Web of Knowledge, China National Knowledge Infrastructure. |
| √ | Search software used, name and version, including special features | We did not employ any search software. EndNote was used to merge retrieved citations and eliminate duplications. |
| √ | Use of hand searching | We hand-searched bibliographies of retrieved papers for additional references. |
| √ | List of citations located and those excluded, including justifications | Details of the literature search process are outlined in the flow chart. The citation list is available upon request. We did not show the exclusion list. |
| √ | Method of addressing articles published in languages other than English | We placed no restrictions on language; Non-Chinese or non-English articles were translated by Google Translate. |
| √ | Method of handling abstracts and unpublished studies | No abstracts or unpublished study was observed. |
| √ | Description of any contact with authors | We contacted with researchers who had conducted relative studies while did not reported the genotype frequency that we needed in their published articles. |
| **Reporting of methods should include** | |  |
| √ | Description of relevance or appropriateness of studies assembled for assessing the hypothesis to be tested | Detailed inclusion and exclusion criteria were described in the methods section. |
| √ | Rationale for selection and coding of data | Data extracted from each of the studies were relevant to the population characteristics, study design, exposure, outcome, and possible effect modifiers of the association. |
| √ | Assessment of confounding factors | Subgroup analysis was performed and we conducted sensitivity analysis by deleting a single study one by one for each time. |
| √ | Assessment of study quality, including blinding of quality assessors; stratification or regression on possible predictors of study results | We developed a quality assessment scale (**Table 1**), which was modified from previous studies. The evaluation mainly focused on representativeness of both cases and controls, specimens of cases for determining genotypes, quality control of genotyping method and sample size. |
| √ | Assessment of heterogeneity | Cochrane’s Q test and the I²index were performed to explore the heterogeneity. |
| √ | Description of statistical methods in sufficient detail to be replicated | Methods of heterogeneity test, quantitative synthesis, assessments of publication bias, sensitivity analyses were detailed in the methods. |
| √ | Provision of appropriate tables and graphics | We provided 1 figure of flow chart to explain the article searching; 1 table for study characteristics; 1 table for pooled analysis; 2 figures of forest plot of all studies; 2 figures of funnel plots to examine publish bias; 1 table for study’ quality assessment; 1 table for sensitivity analysis. |
| **Reporting of results should include** | |  |
| √ | Graph summarizing individual study estimates and overall estimate | Table 3, Figure 2 and Figure 3. |
| √ | Table giving descriptive information for each study included | Table 2 |
| √ | Results of sensitivity testing | Table S1 |
| √ | Indication of statistical uncertainty of findings | 95% confidence intervals were presented with all summary estimates, *P* values and results of sensitivity analyses. |
| **Reporting of discussion should include** | |  |
| √ | Quantitative assessment of bias | Sensitivity analyses indicated that this significant association was stable. |
| √ | Justification for exclusion | We excluded studies that had used different exposure or outcome assessment for the comparison groups, or no control group. |
| √ | Assessment of quality of included studies | We developed a quality assessment scale (**Table 1**), which was modified from previous studies. Most of the included studies were identified as ''moderate-quality'' (13 studies) and ''high-quality'' (12 studies), only three studies identified as "low-quality". |
| **Reporting of conclusions should include** | |  |
| √ | Consideration of alternative explanations for observed results | We discussed that potential unmeasured confounders such as differences in various cancer susceptibilities of various tissues, life style, environmental factor and relative small sample size, might cause different results. |
| √ | Generalization of the conclusions | This meta-analysis suggests that the GSTP1 341C>T polymorphism may contribute to genetic susceptibility to cancer, especially of lung cancer, and in Asian population. |
| √ | Guidance for future research | Further well-designed studies with large sample size in diverse ethnic populations, more types of digestive system cancers along with tissue-specific biochemical, functional and expressional characteristics are required. |
| √ | Disclosure of funding source | No funding supported this study. |
